# The effect of online hygiene information expression on customers' institutional and cognitive trust in tourist accommodation

**DOI:** 10.3389/fpubh.2026.1766094

**Published:** 2026-05-08

**Authors:** BoXi Zhang, Yuxuan Li, Xing Fang, Wei Huang, Qingshen Meng

**Affiliations:** 1 Wuhan University of Technology, Wuhan, China; 2 Huazhong University of Science and Technology Tongji Medical College Tongji Hospital, Wuhan, China

**Keywords:** digital platforms, hotel hygiene information disclosure, structural equation modeling, tourist accommodation, trust

## Abstract

In the digital economy, the ways in which hotels communicate their hygiene standards have become a decisive factor shaping travelers' intention to book, as specific cleaning information—an inherently “invisible service”—is closely related to the credibility perceived through digital platform trust mechanisms. This study examines how hygiene information is conveyed on hotel digital platforms and develops a structural equation model linking “institutional mechanisms → perceived trust → behavioral intention” to investigate the formation pathways of institutional trust and cognitive trust and their mediating roles in users' intention to stay. Based on an online survey of 216 respondents with recent hotel accommodation experience, and using structural equation modeling (SEM) for analysis, the analysis confirms that five institutional mechanisms—standardized cleaning procedures, traceable sanitation pathways, timely feedback responsiveness, information completeness, and hygiene certification—are significantly associated with trust dimensions. The study further introduces “disgust sensitivity toward hygiene” as a moderating variable, showing that highly sensitive users report stronger trust responses when exposed to authoritative hygiene disclosures. Empirical results reveal that cognitive trust is primarily driven by information authenticity and responsive feedback mechanisms, whereas institutional trust hinges on external endorsements and the visualization of cleaning processes; both forms of trust are positively associated with stay intention. The study translates the most influential determinants into actionable design strategies for digital platforms, offering theoretical grounding and practical guidance for optimizing interface and process design in the context of digital hygiene governance.

## 1 Introduction

As digital platforms continue to expand and the demand for short-term rentals increases, platform-based accommodation services—such as online hotel and homestay bookings—have led to a widespread trend of accelerated user decision-making. Hygiene, as the most sensitive yet least “visible” component of service quality (1, 2), has gradually become a central factor shaping users' choices and their trust in digital platforms (3, 4). In nature-based tourism accommodations, visitors' subjective experiences can further magnify hygiene-related distrust (3), especially when cleaning information lacks transparent disclosure, thereby heightening perceived trust risks (4). Consequently, the ability of digital platforms to effectively communicate and disclose hygiene information has become a pivotal mechanism influencing users' trust perceptions and their booking decisions. Traditionally, hotel hygiene encompasses three fundamental components: (1) *environmental hygiene*, referring to the cleanliness and maintenance of guest rooms and public areas; (2) *facility hygiene*, including the disinfection and replacement of linens, bathrooms, and related amenities; and (3) *service hygiene*, which concerns employees' personal hygiene habits and operational standards (5). These practices are typically maintained through standardized on-site operations and manual inspections. However, in the Era of Internet Economy, the hospitality industry has undergone a significant transformation. By leveraging *digital platforms*, hotels have integrated *upgraded hygiene management systems* supported by *third-party certification, co-creation behavior*, and *real-time feedback mechanisms*, thus achieving a new level of digital innovation (6).

Prior studies have shown that digital platforms frequently rely on subjective indicators—such as “cleanliness ratings” or “positive review labels”—in place of standardized, traceable hygiene records. Consequently, users' accommodation choices are heavily influenced by online comments (7), while the absence of unified standards, traceability mechanisms, and participatory frameworks continues to hinder the establishment of systematic trust. Empirical evidence indicates that online reviews serve as one of the most salient external indicators of hotel cleanliness. However, this stream of research presents notable limitations. First, user-generated reviews are often subjective, fragmented, and susceptible to bias, making them insufficient as reliable proxies for actual hygiene conditions. Second, reliance on such indicators overlooks the role of platform-governed institutional mechanisms in shaping trust. As a result, existing studies tend to emphasize outcome-based perceptions rather than process-based transparency, leaving the underlying governance logic of hygiene information largely unexplored.

McKnight identified *trust* as the most pivotal perceptual variable within digital platform service ecosystems (8). Its formation fundamentally depends on *institutional signals* and *information disclosure mechanisms* released by service providers (9). Within hotel digital platforms, trust is not solely related to the form of information disclosure, but is also closely associated with the authenticity, completeness, and consistency of the information relative to users' expectations (10). Building on this perspective, Gefen et al. (9) and Rousseau et al. (11) argued that digital platforms may foster higher levels of user trust through institutional mechanisms characterized by standardized processes, third-party certification, and enhanced transparency and traceability. Such mechanisms effectively reduce users' concerns regarding the “black-box” nature of service procedures and promote greater confidence in the system as a whole. Moreover, disgust sensitivity, rooted in the behavioral immune system theory (12), represents an important individual-difference variable that influences how individuals perceive and respond to potential hygiene threats (13). Individuals with higher disgust sensitivity are more attentive to environmental cues related to contamination and are more likely to rely on institutional safeguards to reduce perceived health risks. In service contexts involving “invisible” processes such as hygiene management, this psychological trait may significantly shape users' trust evaluations and decision-making (44). Therefore, incorporating disgust sensitivity as a moderating variable is theoretically grounded in both behavioral psychology and public health literature (45).

Institutional trust and cognitive trust form two core dimensions in the development of user trust. Institutional trust refers to user confidence in platform-level structures, including formal rules, standardized procedures, and third-party certification that indicate procedural reliability (9). Cognitive trust derives from user assessment of information disclosure, service consistency, and performance records (10, 14). This form of trust is grounded in observable and verifiable evidence of service conduct. Research in e-commerce, healthcare, finance, and public governance identifies these two dimensions as central components in user trust formation (15). Empirical work shows that institutional mechanisms function as the structural basis for the emergence of cognitive trust, and that their joint operation promotes user trust and adoption behavior in digital platforms Given that hygiene safety directly concerns consumers' health and personal wellbeing, investigating how digital platforms foster multidimensional trust through standardized institutional frameworks, transparent procedural disclosure, and third-party certification holds both theoretical and practical significance. Addressing this gap, the present study develops an analytical framework linking institutional mechanisms, trust perception, and behavioral intention in the context of hotel digital platforms. Such inquiry not only deepens understanding of digital trust formation but also provides valuable implications for innovation and governance within the hospitality industry.

Although prior studies have examined trust formation in digital platforms and hospitality contexts, several critical limitations remain. First, existing research predominantly relies on indirect indicators—such as online reviews or perceived cleanliness—rather than systematically analyzing how specific institutional mechanisms (e.g., traceability, certification, and standardized procedures) function as trust-building signals. Second, the interaction between institutional trust and cognitive trust in the context of digital hygiene governance remains underexplored. Third, little attention has been paid to how individual psychological differences, such as disgust sensitivity, shape users' responses to hygiene-related information. Therefore, a clear and systematic investigation of how digital platforms construct multidimensional trust through institutionalized hygiene information disclosure is still lacking.

On the basis of the above, using survey data and empirical analysis, this study aims to systematically investigate how institutionalized hygiene information disclosure on hotel digital platforms influences users' trust formation and behavioral intention. Specifically, this study addresses the following research questions: (1) How do different hygiene-related institutional mechanisms affect institutional trust and cognitive trust? (2) How do these two dimensions of trust influence users' booking intention? (3) How does disgust sensitivity moderate the trust formation process? By addressing these questions, the study seeks to provide both theoretical and practical insights into digital hygiene governance. Therefore, this study makes three main contributions. First, it shifts the focus from outcome-based indicators (e.g., reviews and ratings) to process-based institutional mechanisms, providing a more structured understanding of how hygiene governance is communicated on digital platforms. Second, it integrates institutional trust and cognitive trust into a unified analytical framework, offering new insights into their interaction in the context of digital hospitality. Third, by introducing disgust sensitivity as a moderating variable, the study extends trust research by incorporating individual psychological differences into the analysis of digital hygiene information processing.

## 2 Background and assumptions

### 2.1 Literature review

This study integrates *institutional mechanisms* with the SERVQUAL model and adapts it to the context of *hygiene management on hotel digital platforms*. Building upon this integration, five dimensions are proposed to evaluate the institutional impact on user trust: **Sanitation Task System Perfection**, **Traceability of Cleaning Record**, **Feedback Responsiveness**, **Information Authenticity and Completeness**, and **Hygiene Assurance Certification**. *Institutional Trust* and *Cognitive Trust* are introduced as the core mediating variables. *Institutional Trust* emphasizes users' confidence in rules, standardized procedures, and third-party endorsements, whereas *Cognitive Trust* is grounded in the *transparency* and *authenticity* of disclosed information. Both forms of trust play significant mediating roles in shaping users' *Booking Intention (BI)* toward hotel digital platforms. The theoretical foundation of this study is built upon the Trust in Automation Scale (16), the institutional trust frameworks proposed by Gefen et al. (9) and Rousseau et al. (11), and the perspective on *cognitive insight* articulated by Shamim et al. (17). Together, these provide the conceptual and methodological foundation for analyzing the factors influencing trust in digital platform hygiene information and the mechanisms underlying user behavioral intention.

Existing studies on trust in digital platforms have predominantly relied on user-generated content, such as reviews and ratings, as primary indicators of trust (7). While these studies provide valuable insights, they tend to adopt an outcome-based perspective, overlooking the role of underlying institutional mechanisms. Moreover, prior research often treats trust as a unidimensional construct, neglecting the distinction between institutional trust and cognitive trust. These limitations suggest that current frameworks are insufficient to explain trust formation in contexts where service quality is not directly observable, such as hygiene management in accommodation services. Recent research further emphasizes the importance of institutional governance (18) and transparency in digital environments (19).

In addition, from an interdisciplinary perspective, hygiene perception is closely related to public health and risk perception literature (20). Studies in public health suggest that individuals' behavioral responses to hygiene-related information are strongly influenced by perceived vulnerability to disease, risk perception, and preventive cognition (21). These factors shape how individuals interpret institutional signals and form trust in environments involving potential health risks. Therefore, integrating insights from public health and behavioral science helps enrich the theoretical foundation of this study and provides a more comprehensive understanding of hygiene-related decision-making.

Based on the above review, three key research gaps can be identified. First, existing studies have not sufficiently integrated institutional trust and cognitive trust within a unified framework. Second, there is a lack of research examining how institutional mechanisms shape trust in the context of digital hygiene governance. Third, the role of individual psychological differences, such as disgust sensitivity, remains underexplored. Addressing these gaps is essential for advancing the understanding of trust formation in digital accommodation platforms.

### 2.2 Research hypotheses

On the context of digital platforms, *trust* represents one of the core driving forces behind user decision-making (16). As a formal mechanism of trust assurance, institutional mechanisms are regarded as tools that may help mitigate information asymmetry and service-related risks, and are associated with higher levels of users' trust perception (46). Gefen et al. (9) highlighted that digital platforms strengthen users' perception of legitimacy through formal measures such as *certification systems, credit endorsements*, and *accountability tracing*. The effectiveness of such institutional structures in fostering user trust has been well demonstrated in sharing economy platforms such as Airbnb. Moreover, Bente et al. (22) argued that in service contexts where quality cannot be directly verified, *transparent institutional mechanisms* and *third-party certifications* function as trust proxies and are associated with higher levels of perceived security toward “invisible services.” Therefore, the establishment and implementation of well-designed institutional mechanisms can fundamentally alleviate users' uncertainty, increase their confidence in the hygiene standards promised by digital platforms, and strengthen overall trust formation. Building upon this rationale, the present study further decomposes *institutional mechanisms* into five specific dimensions—**Sanitation Task System Perfection**, **Traceability of Cleaning Record**, **Feedback Responsiveness**, **Information Authenticity and Completeness**, and **Hygiene Assurance Certification, each assumed separately**.

A standardized and well-structured sanitation task system provides clear operational guidelines and evaluation criteria, thereby reducing human arbitrariness and information asymmetry. Pelgander et al. (23) emphasized that in the sharing economy, digital platforms that make operational procedures transparent and standardized allow users to better understand the service process and are associated with higher perceived trust and safety. Such procedural standardization also facilitates supervision and traceability, reinforcing users' expectations of service consistency. From an institutional perspective, standardized processes convey a platform's commitment to hygiene quality through institutional dependence (11), reducing uncertainty and reinforcing users' perception of systemic reliability. Kim and Kim (15) similarly found that public disclosure of standardized procedures is significantly associated with higher levels of institutional trust. According to the SERVQUAL model (24), “standardized operations” are widely considered an important element associated with service reliability and institutional trust. A well-designed sanitation task system thus provides users with a rational institutional basis for trust and mitigates hygiene-related anxiety. From the lens of *cognitive trust*, process standardization and consistent delivery offer observable cues regarding competence, reliability, and predictability (10, 14, 25). Clear and accurate procedural disclosure enhances both the comprehensibility and verifiability of information, thereby strengthening evidence-based cognitive trust (26).

**Hence, the following hypotheses are proposed:**

**H1:** Sanitation Task System Perfection (STP) is positively associated with Institutional Trust (IT).
**H2:** Sanitation Task System Perfection (STP) positively influences Cognitive Trust (CT).

Information traceability and verifiable record-keeping not only strengthen top-down supervision but also play a pivotal role in cultivating user trust. When a digital platform establishes a digital archive for each sanitation operation—recording the full cleaning path, responsible personnel, and time logs—and makes these records accessible to users, trust perception rises substantially. Such traceable documentation aligns with Bachmann's (27) concept of *system trust*, wherein users reduce uncertainty through institutionalized traceability mechanisms that signal procedural integrity and accountability. As Banerjee and Chua (28) argue, a robust “accountability–traceability” system enhances institutional trust by transforming hygiene assurance into a transparent and evidence-based process. Traceability also improves information quality, source credibility, and diagnosticity, enabling users to form rational, evidence-driven judgments regarding hygiene reliability (16, 29). Within *credence service* contexts, traceability frameworks have been empirically shown to increase consumers' confidence in certified claims, thereby strengthening *cognitive trust* (30). In the context of hotel digital platforms, audit and accountability systems that allow users to view real-time cleaning information—such as today's room sanitation records—cultivate a tangible sense of control and reliability, greatly reinforcing hygiene-related trust.

**Hence, the following hypotheses are proposed:**

**H3:** Traceability of Cleaning Record (TCR) positively influences Institutional Trust (IT).
**H4:** Traceability of Cleaning Record (TCR) positively influences Cognitive Trust (CT).

Timely feedback mechanisms, when coupled with concrete remedial and punitive measures, are critical to reducing user dissatisfaction and uncertainty—thus serving as a vital pathway to strengthening *institutional trust*. Lin et al. (31) demonstrated that when users raise concerns or complaints about poor hygiene conditions, a digital platform's prompt response, corrective action, and enforcement of accountability significantly accelerate the recovery of *cognitive trust*. From the perspective of institutional mechanism theory, effective feedback not only provides a procedural avenue for problem resolution but also conveys the platform's sense of responsibility, service competence, and commitment to continuous improvement (32) further found that sincere apologies can enhance both affective and cognitive trust, consequently increasing users' willingness to repurchase and recommend services. While apologies tend to mitigate perceived risk, they may not directly influence purchase intention; rather, it is the timeliness and efficacy of feedback that shape users' trust evaluations (47). Conversely, delayed or absent feedback amplifies users' negative expectations regarding hygiene reliability and can even exacerbate trust crises. Therefore, the integration of a responsive feedback mechanism with tangible remedial actions represents a crucial strategy for fostering hygiene-related trust in digital hospitality platforms.

**Hence, the following hypotheses are proposed:**

**H5:** Feedback Responsiveness (FR) positively influences Institutional Trust (IT).
**H6:** Feedback Responsiveness (FR) positively influences Cognitive Trust (CT).

The authenticity of sanitation information relies on standardized and structured presentation, which may contribute to higher levels of users' trust in a digital platform's hygiene commitments. Empirical evidence suggests that the *authenticity* and *completeness* of disclosed information significantly enhance user trust in digital platforms, as detailed and transparent disclosure reduces perceived risk and strengthens confidence in decision-making (33). Compared with vague or promotional statements, *data-driven and verifiable hygiene information*—such as explicit cleaning times, responsible personnel, and specific disinfection measures—elicits stronger trust responses from users. Banerjee and Chua (28) further argued that standardized data presentation minimizes users' cognitive load when interpreting information, enabling more intuitive judgments about service reliability. Theoretically, such mechanisms improve the *objectivity* of information disclosure—providing “evidence-based transparency” that strengthens the rational foundation of trust. Prior studies consistently emphasize that the *authenticity* and *completeness* of information are core determinants of user trust in digital platforms (16, 22). Consequently, transparent, factual, and standardized information communication fosters users' belief that digital platforms do not conceal facts, thereby enhancing both institutional and cognitive dimensions of hygiene-related trust.

**Hence, the following hypotheses are proposed:**

**H7:** Information Authenticity and Completeness (IAC) is positively associated with Institutional Trust (IT).
**H8:** Information Authenticity and Completeness (IAC) is positively associated with Cognitive Trust (CT).

Third-party certification of cleaning equipment and materials provides authoritative validation, effectively mitigating users' skepticism toward the invisible aspects of hotel hygiene. Compared with self-declared claims made by digital platforms, *transparent certification* conducted by independent institutions substantially enhances the *credibility* of sanitation information, instilling stronger trust and a sense of security among users (34). It has been found that when platforms or hotels disclose verifiable cleaning certificates or the regulatory compliance of disinfectants, users' perceived risk decreases markedly, while their confidence in hygiene quality rises correspondingly. From a theoretical standpoint, third-party certification serves as an *external institutional safeguard* that embodies neutrality and objectivity—thereby reinforcing the dimension of *institutional trust* described in trust theory (16). Transparent hygiene certification thus plays a crucial role in enhancing users' overall trust in a hotel's cleanliness and hygiene management.

**Hence, the following hypotheses are proposed:**

**H9:** Hygiene Assurance Certification (HAC) positively influences Institutional Trust (IT).
**H10:** Hygiene Assurance Certification (HAC) is positively associated with Cognitive Trust (CT).

The *manner and quality* of information disclosure directly shape users' perceived safety and booking intentions. In digital accommodation contexts, users tend to trust *structured and standardized hygiene information* rather than promotional narratives. Sun et al. (34) demonstrated that the *completeness* of hygiene information and the *authenticity* of online cleanliness ratings exert significant effects on consumers' booking decisions. Similarly, Li and Wang (35) highlighted that when digital platforms adopt standardized mechanisms for hygiene disclosure—such as specifying cleaning time, responsible personnel, certification type, and disinfection procedures—users' perceived risk decreases substantially, encouraging booking behavior. Standardized and factual disclosure reduces users' cognitive effort in interpreting information, while improving its *verifiability* and *reliability*. This aligns with the *tangibility* and *reliability* dimensions of the SERVQUAL model, both of which positively affect consumers' behavioral intentions. Therefore, presenting hygiene information in a standardized and institutionalized form not only enhances users' cognitive transparency but also strengthens their behavioral willingness to book accommodations.

**Hence, the following hypothesis is proposed:**

**H11:** Information Authenticity and Completeness (IAC) directly enhance Booking Intention (BI).

Third-party hygiene certification functions as an authoritative endorsement that enhances the *verifiability* and *transparency* of hygiene information, reduces perceived risk, and consequently strengthens both *institutional trust* and *cognitive trust*, which in turn fosters booking intention. Early studies incorporating trust and risk into online consumer behavior models identified trust as a decisive driving factor (9, 32) further proposed a trust-centered framework for consumer decision-making, emphasizing the mediating role of trust in online purchase behavior. Similarly, Xie et al. (7) found that the credibility of brand image and informational endorsement significantly influences both trust and behavioral intention in online accommodation settings. Synthesizing these findings, within hotel digital platforms, *hygiene certification* and *transparent information disclosure* serve as institutional guarantees that effectively mitigate information asymmetry and enhance user trust.

**Hence, the following hypothesis is proposed:**

**H12:** Hygiene Assurance Certification (HAC) is positively associated with Booking Intention (BI).

Trust has been repeatedly verified as a key determinant influencing users' behavioral intentions—such as platform choice, recommendation willingness, and repurchase behavior—across digital service environments (9). In the context of hotel digital platforms, hygiene safety represents a highly sensitive service attribute. When users develop strong trust in a hotel's hygiene management, their anxiety regarding potential post-stay risks or discomfort diminishes, which enhances decisiveness in booking. Cheng et al. (36) found that within the sharing accommodation sector, higher trust in the authenticity of cleanliness standards and execution increases users' likelihood of initial booking, repeat purchases, and active recommendations. Moreover, highly trusting users tend to show greater tolerance and positive word-of-mouth when service failures occur. Therefore, higher levels of users' trust in hygiene management are associated with stronger behavioral intentions, including booking, repurchasing, and recommending.

**Hence, the following hypotheses are proposed:**

**H13:** Institutional Trust (IT) is positively associated with Booking Intention (BI).
**H14:** Cognitive Trust (CT) is positively associated with Booking Intention (BI).

Disgust sensitivity (36) can be conceptualized as a stable individual difference along the dimension of pathogen-related threat (e.g., pathogen disgust or perceived vulnerability to disease). This trait systematically shapes individuals' attention to, processing of, and responsiveness to hygiene-relevant cues. According to behavioral immune system theory, individuals with higher pathogen disgust or greater perceived vulnerability respond more sensitively to disease-related environmental cues and rely more heavily on institutional signals and evidential safeguards—such as standardized procedures, traceable records, and third-party certifications—to reduce uncertainty and form trust judgments that guide avoidance or approach behaviors (13, 37).

In service and tourism contexts, health-risk perception and health awareness not only exert direct effects on trust and intention but also amplify the influence of hygiene and safety measures on user evaluations. Hotel cleaning and health-safety practices increase brand trust and loyalty by reducing perceived health risks, with stronger effects among individuals who perceive higher vulnerability (38). Research on travel intention further shows that health-risk perception alters individuals' reliance on and responsiveness to protective cues, demonstrating a significant moderating role (39, 40).

**Hence, the following hypotheses are proposed:**

**H15:** Disgust sensitivity (DS) moderates the strength of institutional trust (IT).
**H16:** Disgust sensitivity (DS) moderates the strength of cognitive trust (CT).

**Figure 1:**
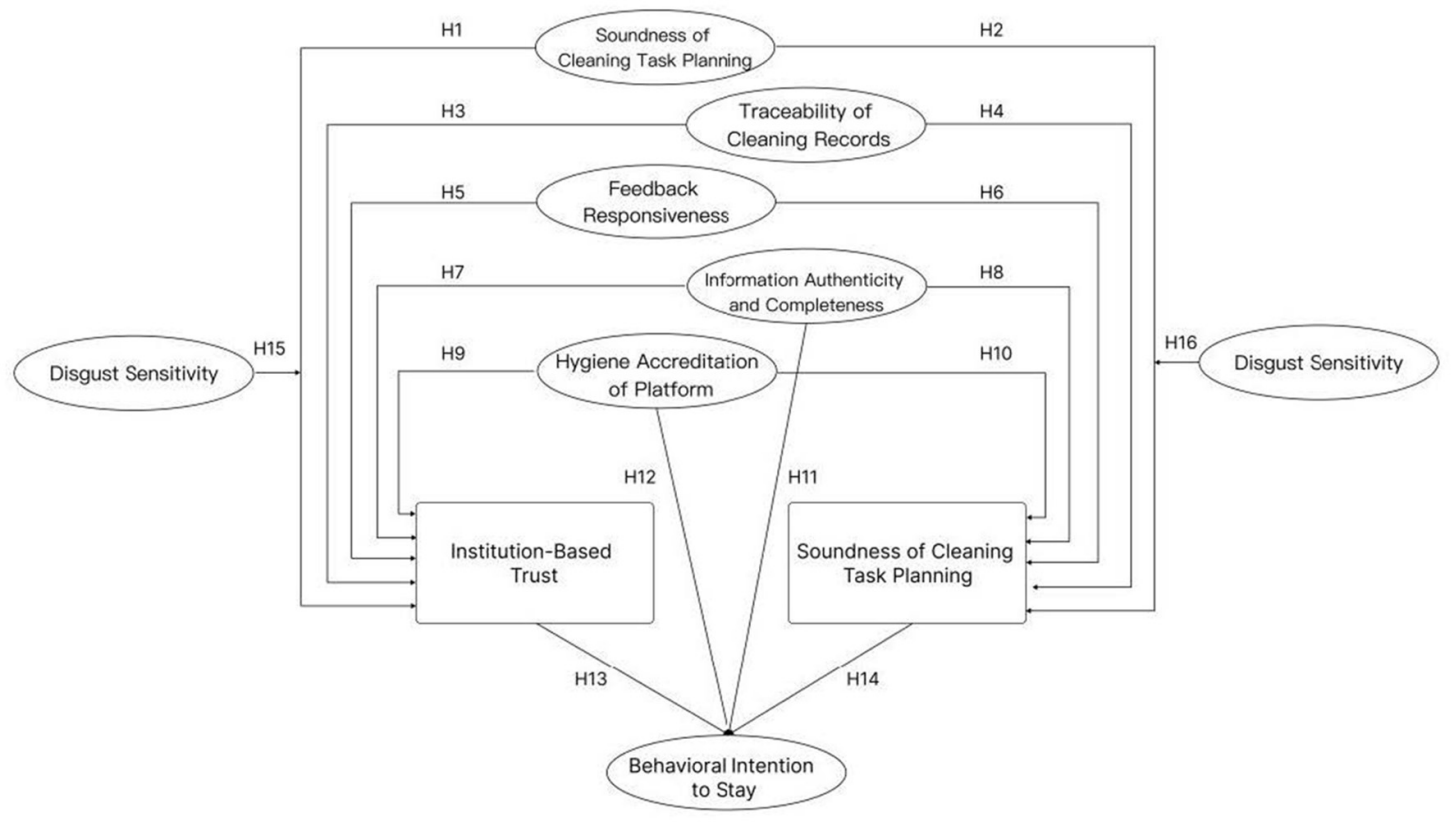
Conceptual framework.

Therefore, the model path is shown in Figure 1.

## 3 Data analysis and research results

### 3.1 Sample and data description

This study employed an online survey method to collect data and empirically test the proposed research model and hypotheses. The questionnaire was developed based on valid and reliable measurement items from existing literature. Specifically, the measurement items were adapted from established scales in studies on digital trust and service quality, and were refined to reflect the context of hotel hygiene information disclosure on digital platforms (29, 41). Before the formal survey, the questionnaire underwent a small-scale pilot test to ensure clarity and contextual suitability of the items.

The formal survey was conducted between April and September 2025 among participants who had prior hotel accommodation experience. Participation in the survey was entirely voluntary. The questionnaire was distributed via an online link, and respondents could complete it using either mobile or desktop devices. All measurement items were assessed using a seven-point Likert scale (1 = strongly disagree, 7 = strongly agree), capturing respondents' perceptions and attitudes toward hotel cleanliness mechanisms.

Participants were recruited through online distribution channels, and responses were carefully screened to ensure that only valid questionnaires completed by users with relevant platform experience were included in the final dataset. This procedure helped improve the reliability and relevance of the collected data. The final sample size of 216 respondents meets commonly recommended thresholds for structural equation modeling (SEM), which typically suggest a minimum of around 200 observations to ensure stable parameter estimation. Nevertheless, because the survey relied on voluntary online participation, a certain degree of self-selection bias may exist. Therefore, this limitation should be considered when interpreting the generalizability of the findings.

Table 1 presents the demographic characteristics of the respondents. In terms of gender, 46.8% were male (*n* = 101) and 53.2% were female (*n* = 115), indicating a relatively balanced gender distribution. Regarding age, 32.9% were aged 18–25 (*n* = 71), 38.4% were aged 26–35 (*n* = 83), 20.4% were aged 36–45 (*n* = 44), and 8.3% were aged 46 or above (*n* = 18). In addition, participants reported different frequencies of hotel stays during the past year: 41.7% (*n* = 90) stayed fewer than three times, 33.3% (*n* = 72) stayed four to six times, and 25.0% (*n* = 54) reported seven or more stays.

**Table 1:** Descriptive statistics of respondent profile (*N* = 216).

Variable category	Group	Sample size (*n*)	Percentage (%)
Gender	Male	101	46.8
	Female	115	53.2
Age	18–25 years	71	32.9
	26–35 years	83	38.4
	36–45 years	44	20.4
	46 years and above	18	8.3
Online hotel booking frequency	Less than 3 times	90	41.7
	4–6 times	72	33.3
	7 times or more	54	25.0

Structural equation modeling (SEM) was employed because it allows the simultaneous examination of relationships among multiple latent constructs and the testing of mediating and moderating effects within a unified analytical framework. This approach is particularly suitable for the present study, which investigates the complex relationships between institutional mechanisms, multidimensional trust, and behavioral intention.

### 3.2 Reliability and validity analysis

To assess the potential impact of common method bias, Harman's single-factor test was conducted. The results indicated that the first factor accounted for less than 50% of the total variance, suggesting that common method bias is unlikely to pose a serious threat to the validity of the results. Additionally, procedural remedies such as anonymous responses and clear questionnaire instructions were adopted to reduce potential common method bias.

Cronbach's α values for all constructs ranged from 0.677 to 0.950. With the exception of Behavioral Intention (BI), which was slightly below 0.7, all other constructs exceeded 0.8, indicating strong internal consistency. The Composite Reliability (CR) values were all greater than 0.7, confirming satisfactory convergent reliability of the latent constructs. Furthermore, all Average Variance Extracted (AVE) values exceeded 0.5, demonstrating adequate convergent validity (see Table 2).

**Table 2:** Reliability and validity analysis.

Factor	Cronbach's α	Composite reliability	AVE
STP	0.823	0.738	0.738
TCR	0.824	0.74	0.74
FR	0.87	0.793	0.793
IAC	0.854	0.775	0.775
HAC	0.826	0.743	0.743
IT	0.941	0.895	0.895
CT	0.95	0.911	0.911
BI	0.677	0.611	0.611
DS	0.876	0.802	0.802

The discriminant validity matrix (see Table 3) followed the Fornell–Larcker criterion, where the square root of each construct's AVE (on the diagonal) was higher than any inter-construct correlation in the corresponding column. This result confirmed strong discriminant validity among all constructs. In addition, robustness and bias tests were conducted.

**Table 3:** Discriminant validity analysis.

Factor	STP	TCR	FR	IAC	HAC	IT	CT	BI	DS
STP	**0.859**								
TCR		**0.86**							
FR			**0.891**						
IAC				**0.88**					
HAC					**0.862**				
IT						**0.946**			
CT							**0.954**		
BI								**0.782**	
DS									**0.896**

Bold diagonal values are the square root of the average variance extracted (AVE) for each construct, used to assess discriminant validity.

To ensure the robustness of the results, additional analyses were conducted. Alternative model specifications were tested to verify the stability of the structural relationships. Furthermore, common method bias was assessed using Harman's single-factor test, and the results indicated that no single factor accounted for the majority of the variance, suggesting that common method bias was not a serious concern in this study.

### 3.3 Main path analysis

**Figure 2:**
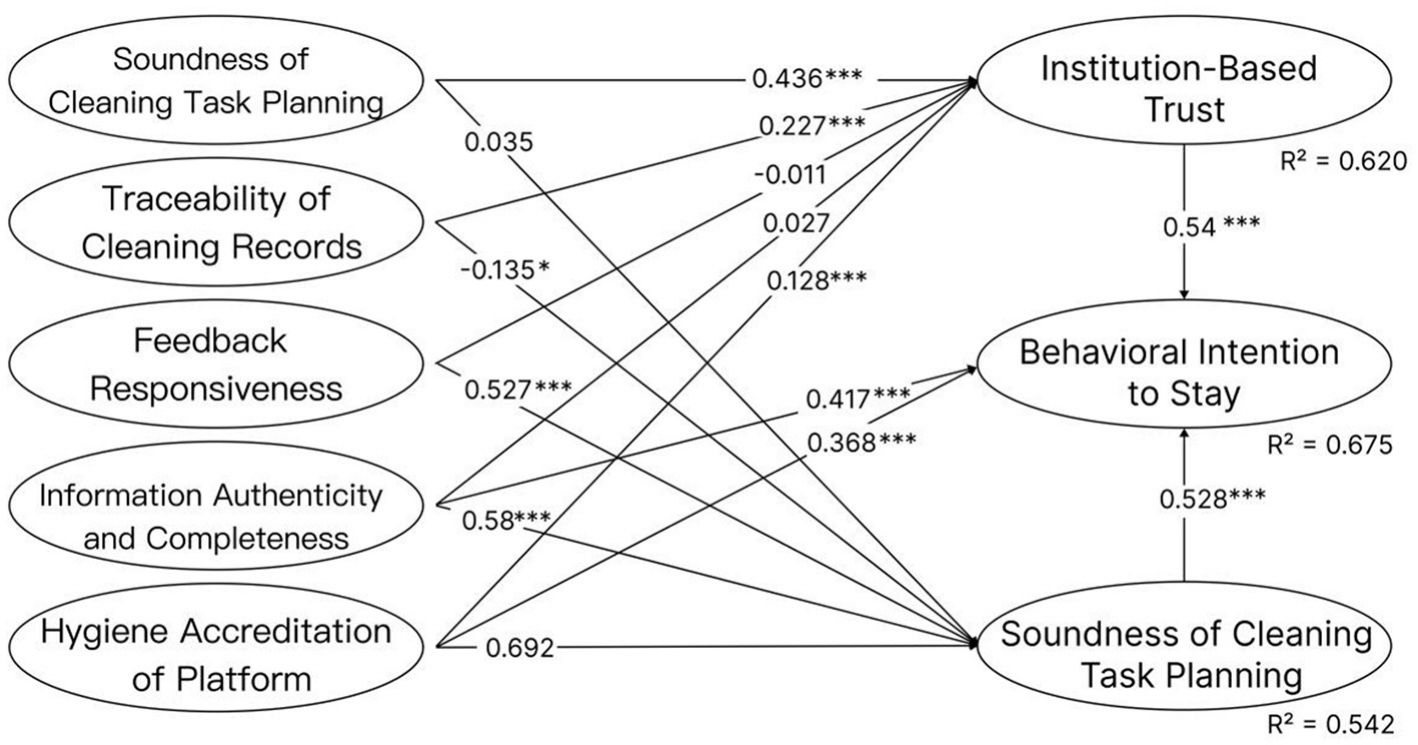
Main effect hypothesis path. Significance levels: *p < 0.05,0**p < 0.001; paths with no symbol indicate0*p* ≥ 0.050and are not statistically significant.

To approximate the structural equation model (SEM) relationships, regression analyses were conducted to estimate the standardized path coefficients. The main significant paths and their statistical indicators—including standardized coefficients (β), *t-values*, and *p-values*—are summarized in Table 4 and Figure 2, covering all hypothesized relationships (H1–H14).

**Table 4:** Main path analysis.

Hypothesis	Path	β	*t*	*p*	Significance
H1	STP → IT	0.436	7.097	0.0	^***^
H2	STP → CT	0.035	0.518	0.6053	
H3	TCR → IT	0.227	3.403	0.0008	^***^
H4	TCR → CT	−0.135	−1.999	0.0468	^*^
H5	FR → IT	−0.011	−0.158	0.8749	
H6	FR → CT	0.527	9.065	0.0	^***^
H7	IAC → IT	0.027	0.401	0.689	
H8	IAC → CT	0.58	10.428	0.0	^***^
H9	HAC → IT	0.692	14.03	0.0	^***^
H10	HAC → CT	0.128	1.888	0.0603	
H11	IAC → BI	0.417	6.71	0.0	^***^
H12	HAC → BI	0.368	5.796	0.0	^***^
H13	IT → BI	0.54	9.381	0.0	^***^
H14	CT → BI	0.528	9.088	0.0	^***^

Significance levels: ^*^p < 0.05, ^***^p < 0.001; paths with no symbol indicate *p* ≥ 0.05 and are not statistically significant.

The results reveal that **system transparency (STP)**, **traceability (TCR)**, **information authenticity and completeness (IAC)**, and **hygiene assurance certification (HAC)** are significantly associated with higher levels of user trust. The results further suggest that trust plays a mediating role between institutional mechanisms and behavioral intention. Institutional signals influence users' booking decisions indirectly through both institutional trust (IT) and cognitive trust (CT), highlighting the importance of trust as a psychological transmission mechanism. In practical terms, this pattern suggests that when digital platforms provide clearer hygiene procedures and transparent sanitation information, users may perceive the platform as more trustworthy in managing hygiene risks. This indicates that visible hygiene governance mechanisms may serve as important signals that reduce uncertainty for potential hotel guests.

### 3.4 Adjustment path analysis

**Figure 3:**
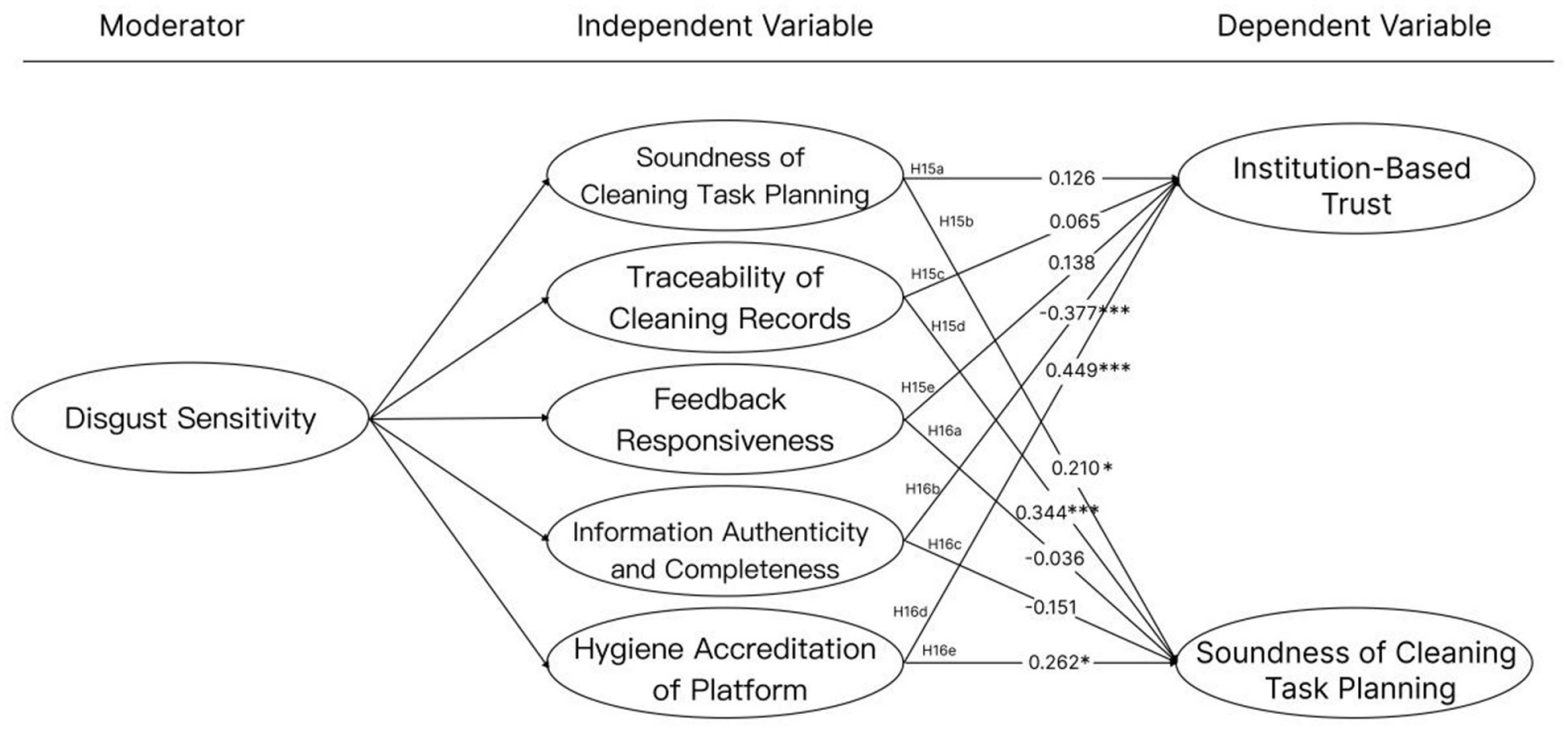
Adjustment path analysis. Significance levels: *p < 0.05,0**p < 0.001; paths with no symbol indicate0*p* ≥ 0.050and are not statistically significant.

Table 5 and Figure 3 adjustment path analysis present the regression outcomes for all hypothesized moderation paths (**H15a–H16e**), including standardized coefficients (β), *t*-values, and *p*-values, with significance levels indicated. These results provide a comprehensive overview of the empirical support for each theoretical pathway. The findings reveal that users with **high disgust sensitivity (DS)** rely more heavily on **digital platform hygiene certification (HAC)** and **information authenticity and completeness (IAC)** to establish both **institutional trust (IT)** and **cognitive trust (CT)**. Particularly regarding institutional trust, highly sensitive users show a stronger trust response to certified information, indicating that external verification mechanisms play a crucial role in reducing perceived uncertainty among this group.

**Table 5:** Adjustment path analysis.

Hypothesis	Path	β	*t*	*p*	Significance
H15a	STP × DS → IT	0.126	1.426	0.1554	
H15b	TCR × DS → IT	0.065	0.855	0.3935	
H15c	FR × DS → IT	0.138	1.576	0.1164	
H15d	IAC × DS → IT	−0.377	−3.988	0.0001	^***^
H15e	HAC × DS → IT	0.449	6.008	0.0000	^***^
H16a	IAC × DS → CT	0.210	2.593	0.0102	^*^
H16b	FR × DS → CT	0.344	4.844	0.0000	^***^
H16c	TCR × DS → CT	−0.036	−0.454	0.6504	
H16d	STP × DS → CT	−0.151	−1.493	0.1369	
H16e	HAC × DS → CT	−0.262	−2.275	0.0239	^*^

Significance levels: ^*^p < 0.05, ^***^p < 0.001; paths with no symbol indicate *p* ≥ 0.05 and are not statistically significant.

**Figure 4:**
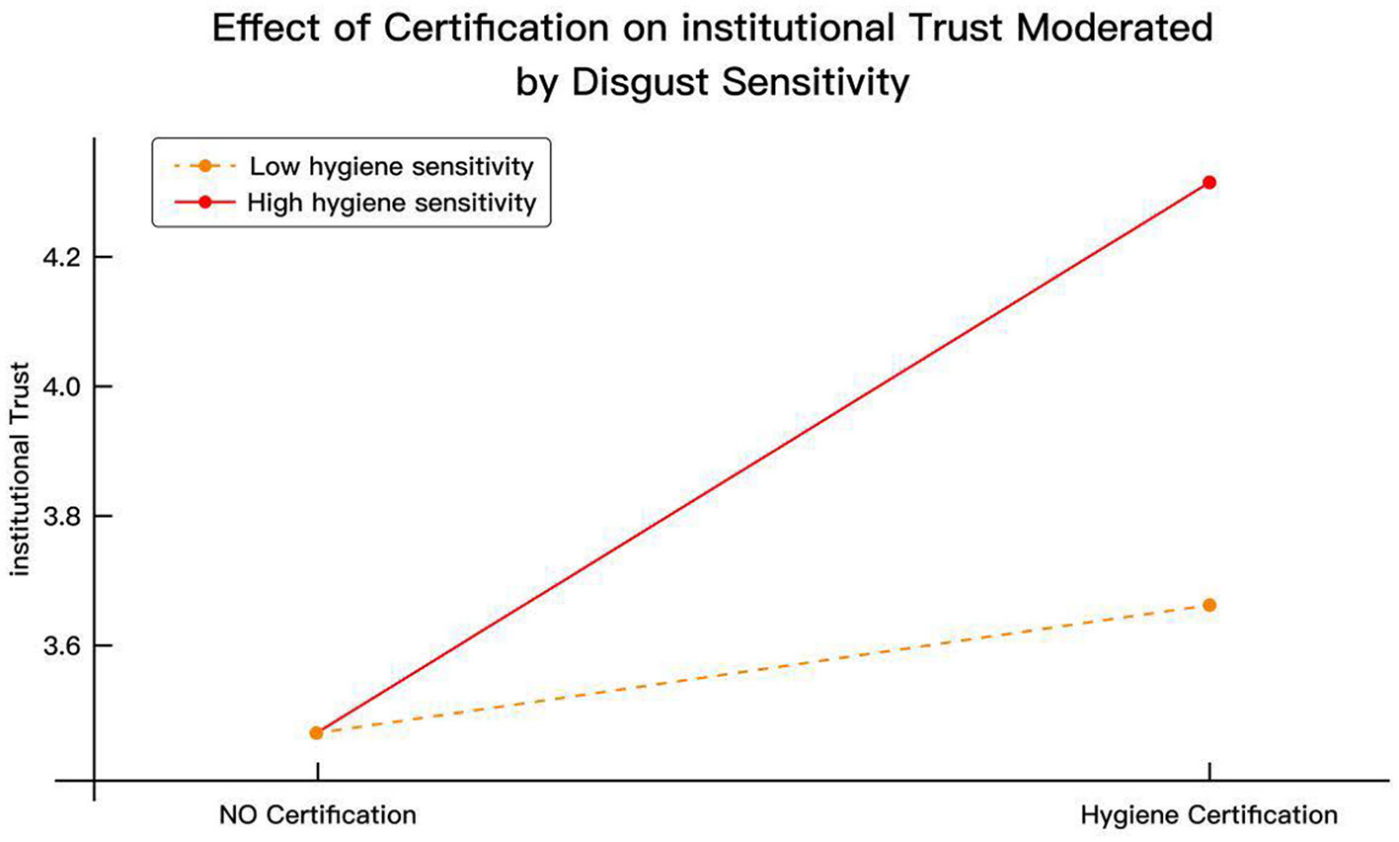
Effect of certification on institutional trust moderated by disgust sensitivity.

Figure 4 illustrates how disgust sensitivity moderates the effect of hygiene certification (HAC) on institutional trust (IT). When no hygiene certification was provided, both high- and low-sensitivity users reported similar levels of institutional trust (mean = 3.45). However, once hygiene certification information was displayed, trust levels diverged significantly: institutional trust among high-sensitivity users increased sharply to 4.35 (Δ = +0.90), whereas low-sensitivity users showed only a modest rise to 3.65 (Δ = +0.20). This supports the significance of H15e (β = 0.449, *p* < 0.001) and indicates that users with high pathogen sensitivity exhibit a stronger positive response to institutional assurances, reinforcing the moderating effect of DS in governance-based trust formation.

**Figure 5:**
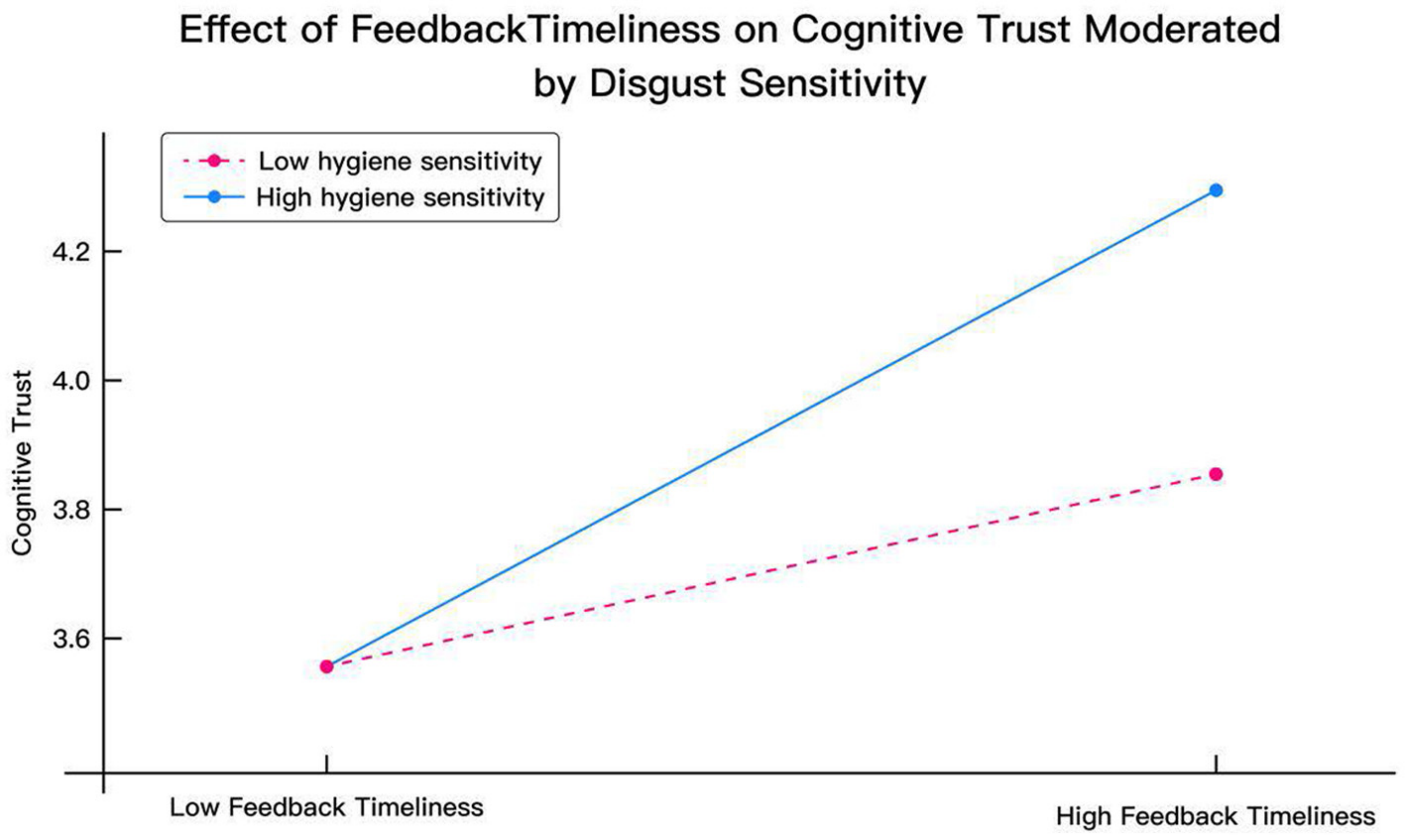
Effect of feedback timeless on cognitive trust moderated by disgust sensitivity.

Figure 5 demonstrates the moderating role of disgust sensitivity in the relationship between feedback responsiveness (FR) and cognitive trust (CT). Under conditions of low responsiveness, both sensitivity groups showed similarly low levels of cognitive trust (mean = 3.55). However, when the platform's response became more prompt, cognitive trust among high-sensitivity users rose substantially to 4.30, while low-sensitivity users exhibited a smaller increase to 3.85. The interaction term in this path (H16b) was significant (β = 0.344, *p* < 0.001), indicating that high-DS users are particularly responsive to micro-level service signals, such as prompt feedback, which they interpret as evidence of reliability and attentiveness.

Overall, most model paths reached statistical significance (*p* < 0.05), supporting the proposed theoretical framework. Within the **institutional trust (IT)** dimension, **hygiene assurance certification (HAC**, **β** **=** **0.692)**, **system transparency (STP**, **β** **=** **0.436)**, and **traceable cleaning records (TCR**, **β** **=** **0.227)** emerged as significant positive predictors, indicating that users tend to rely on visible institutional mechanisms—such as certification and transparent procedures—when evaluating the credibility of hygiene governance on digital platforms, suggesting that institutional trust primarily derives from the platform's ability to demonstrate structured and verifiable hygiene governance.

In the **cognitive trust (CT)** dimension, **information authenticity and completeness (IAC**, **β** **=** **0.580)** and **feedback responsiveness (FR**, **β** **=** **0.527)** were the strongest predictors, while **traceability (TCR**, β **=** **−0.135)** exerted a weaker, negative effect. This implies that users' cognitive trust depends more on the **perceived transparency and timeliness** of the platform's hygiene communication than on retrospective traceability records.

Finally, in predicting **behavioral intention (BI)**, both trust constructs exerted significant positive influences—**institutional trust (β** **=** **0.540)** and **cognitive trust (β** **=** **0.528)**—with nearly equivalent explanatory power, suggesting that both institutional safeguards and information-based trust perceptions may play similarly important roles in shaping users' accommodation decisions. These results affirm the study's core theoretical logic.

After incorporating control variables (i.e., gender, age, and hotel booking frequency) into the baseline model, the significance and directional signs of the core hypothesized paths remained consistent with the main results, suggesting that our findings are robust.

## 4 Discussion

The empirical results support prior research by showing that standardized hygiene management and transparent information disclosure are positively associated with higher levels of user trust and booking intention within the sampled population. The findings correspond with studies identifying trust as a determinant of behavioral intention, including work by Gefen et al. (9) and McKnight et al. (16) on institutional trust in digital platforms and by Banerjee and Chua (28) on the role of institutional processes and certification in reducing uncertainty under conditions of unverifiable service quality. The data further indicate that standardized hygiene protocols, accountability traceability, and third-party certification appear to reduce perceived information asymmetry and service opacity, which in turn are associated with higher levels of user trust.

Moreover, this study presents a systematic analysis of trust formation paths and clarifies the role of cognitive trust in digital hygiene service contexts. In contrast to research that infers trust from user-generated content such as reviews or ratings, the analysis in this study centers on the structural logic of institutional mechanisms. In digital accommodation settings, hygiene standardization and transparent disclosure reduce uncertainty and risk, leading to the formation of institutional trust and cognitive trust and, consequently, to increased booking intention (16).

More specifically, hygiene standards, procedural regulations, accountability mechanisms, and third-party certifications provide users with verifiable cues and structural assurances, which are statistically associated with stronger behavioral intentions in the sampled data (8, 35). On this foundation, several distinct findings emerge:

1. Asymmetry in trust pathways—Feedback responsiveness exerts a stronger marginal effect on cognitive trust than on institutional trust, whereas third-party hygiene certification more strongly reinforces institutional trust than cognitive trust.
2. Information authenticity and completeness (IAC) represent the most powerful predictor of cognitive trust, while traceability (TCR) and standardized procedures (STP) primarily strengthen institutional trust.
3. These asymmetrical effects conform to theoretical expectations: cognitive trust depends more on verifiable, concrete information cues, whereas institutional trust is grounded in external assurances and procedural consistency (10, 15, 35).

Accordingly, digital platforms should differentiate between the leverage points of cognitive trust and institutional trust when designing governance and communication strategies. In practical terms, this means emphasizing detailed, transparent, and verifiable hygiene information to strengthen cognitive trust, while simultaneously maintaining systematic certification and process standardization to consolidate institutional trust.

Notably, while Traceable Cleaning Records (TCR) exerted a positive effect on Institutional Trust (β = 0.227), it demonstrated a significant negative effect on Cognitive Trust (β = −0.135, *p* = 0.0468). This paradox suggests that although technological traceability is positively associated with institutional trust in digital platforms, it may simultaneously be interpreted by users as a form of surveillance or a signal of potential risk, thereby undermining their subjective sense of cognitive trust. This finding echoes Ellis et al. (42), who argued that excessive technological oversight can inadvertently erode user comfort and perceived autonomy. Thus, while institutional governance mechanisms can enhance transparency, their design must carefully consider users' psychological receptivity, ensuring that “technological empowerment” does not evolve into “trust attenuation” (28).

Additionally, the empirical analysis in this study suggests the presence of a potential “trust threshold” associated with the completeness of evidence disclosure. When platforms provide only hygiene ratings without transparent accountability chains or process documentation, the data indicate that users' reported trust and behavioral intentions do not increase significantly within the sampled context. Conversely, ambiguous or unverifiable disclosures can even produce trust depreciation or diminishing marginal effects, as users perceive inconsistency between declared and verifiable information. This aligns with the observations of Filieri (41) and Beldad et al. (29), who found that fragmented or vague information disclosures fail to sustain stable cognitive trust or behavioral conversion. In other words, the findings suggest that users may respond not only to the presence of hygiene information but also to the perceived integrity and verifiability of the disclosed evidence. Compared with these earlier studies, which largely examined transparency in general e-commerce settings, the present findings highlight the heightened importance of evidence completeness in hospitality contexts, where hygiene management is inherently invisible and must therefore be communicated through structured informational signals.

This study incorporated Disgust Sensitivity (DS) as a moderating variable within the institutional mechanism–trust model to examine how individual psychological differences may influence trust formation. The results indicate that users with higher hygiene sensitivity report relatively stronger trust responses and booking intentions when exposed to structured hygiene information. This supports theoretical perspectives by Curtis and Biran (43) who highlighted that individual differences in pathogen sensitivity systematically shape trust judgments and behavioral choices. Nevertheless, compared with earlier studies that examined disgust sensitivity mainly in health behavior or risk perception contexts, the present findings extend this perspective to digital hospitality platforms, suggesting that hygiene-related emotional responses may shape how users interpret platform governance signals (44). The moderation analysis further confirmed that DS significantly moderates the paths HAC → IT, FR → CT, and IAC → CT, suggesting that, within the sampled data, users with higher hygiene sensitivity tend to report relatively stronger trust responses under conditions of structured hygiene information disclosure, and that trust may play a more prominent mediating role in shaping their behavioral intentions. These patterns suggest that individual psychological predispositions may shape how institutional transparency mechanisms are cognitively processed in digital accommodation environments.

However, not all institutional mechanisms function equivalently across user groups. For instance, the interaction terms STP → IT and TCR → CT were not significant, implying a possible “mechanism–cognition mismatch.”

One possible explanation is that users may associate system transparency primarily with operational clarity rather than with institutional reliability, while traceability mechanisms may be perceived as technical monitoring tools rather than as cues for cognitive trust formation. Compared with studies conducted in broader digital governance contexts, the present findings suggest that trust formation in hospitality platforms may require more closely aligned informational signals that correspond to users' specific hygiene concerns and evaluation heuristics. Consequently, platform governance strategies may benefit from adopting differentiated communication approaches that account for varying user perceptions and sensitivity profiles.

Overall, the data indicate a stronger statistical association between institutional mechanisms and trust perceptions among high-sensitivity users, whereas low-sensitivity users display relative “trust inertia” and weaker behavioral conversion. This suggests that digital platforms should emphasize segmented communication and user education in hygiene-related information strategies to improve overall trust and behavioral outcomes. At the same time, although the moderating role of DS proved robust, not all institutional interventions produce universal enhancement effects, highlighting the contextual limits of institutional trust in digital service environments. Because the study relies on cross-sectional survey data, the reported relationships should be interpreted as associative patterns rather than definitive causal effects. Furthermore, the moderating effects identified in the analysis should be considered exploratory, as subgroup sizes are relatively limited and the analysis aims to identify indicative patterns rather than definitive causal differences.

Theoretically, this study contributes to the literature by extending trust theory from outcome-based evaluations to process-based institutional mechanisms in digital hygiene governance. Unlike prior studies that primarily rely on user-generated reviews, this research demonstrates that structured institutional signals—such as certification, traceability, and standardized procedures—serve as fundamental drivers of trust formation. Furthermore, by integrating institutional trust and cognitive trust into a unified framework, the study reveals their asymmetric roles in shaping behavioral intention. Finally, the incorporation of disgust sensitivity introduces a psychological dimension, linking digital trust formation with behavioral immune system theory.

One of design's central challenges is converting abstract theory into concrete, perceivable interactions on digital platforms. Drawing on the empirical findings—particularly the four most influential variables [Hygiene Assurance Certification **(HAC)**, Information Authenticity and Completeness **(IAC)**, Feedback Responsiveness **(FR)**, and the moderator Disgust Sensitivity **(DS)**]—we propose four targeted design strategies to enable effective translation from platform hygiene governance to user trust and behavioral conversion. From a public health perspective, digital platforms play an increasingly important role in communicating hygiene risks and preventive measures. Transparent hygiene information not only reduces perceived uncertainty but also contributes to public health awareness and risk mitigation. Therefore, digital hygiene governance can be understood as an extension of public health communication in platform-based service environments.

1. **Certification visibility: visualizing HAC**
  HAC emerged as the strongest driver of *Institutional Trust (IT)* and is highly sensitive to users' DS. Platforms should therefore embed authoritative certification signals directly into the decision flow. Practical UI patterns include badge systems, real-time certification status, tiered certification labels, and clickable certification details. Structuring certifications visually reduces uncertainty and foregrounds institutional endorsement at the moment of choice.
2. **Structured hygiene disclosure: standardizing IAC**
  IAC is the sole variable most strongly driving *Cognitive Trust (CT)* and Booking Intention (BI). Users prioritize hygiene information that is **authentic, specific, and verifiable**. Implement standardized disclosure templates—“task cards” that list responsible staff, cleaning timestamp, cleaned areas, disinfectants and procedures—rather than vague marketing copy. Such templates increase transparency, lower interpretation cost, and provide audit-ready evidence that supports evidence-based cognitive judgments.
3. **Visible remediation workflow: surfacing FR**
  FR significantly affects CT, with amplified effects for high-DS users. Introduce visible remediation affordances: issue progress bars for complaint handling, avatars and names of responsible personnel, stepwise status updates, and push/email reminders of outcomes. These micro-interaction cues increase users' perceived control and accelerate trust repair by demonstrating competence, accountability, and responsiveness.
4. **Sensitivity-aware interfaces: DS-based layering**
  DS moderates multiple trust pathways—especially HAC → IT and IAC → CT—so platforms should detect or allow users to self-declare hygiene sensitivity and deliver tiered information accordingly. For high-DS users, default displays should surface detailed cleaning logs, certification evidence, and fast-response channels; for low-DS users, present a streamlined summary to avoid cognitive overload. This segmentation improves perceived fit, reduces unnecessary friction, and realizes “adaptive trust” through personalized information density.

**Figure 6:**
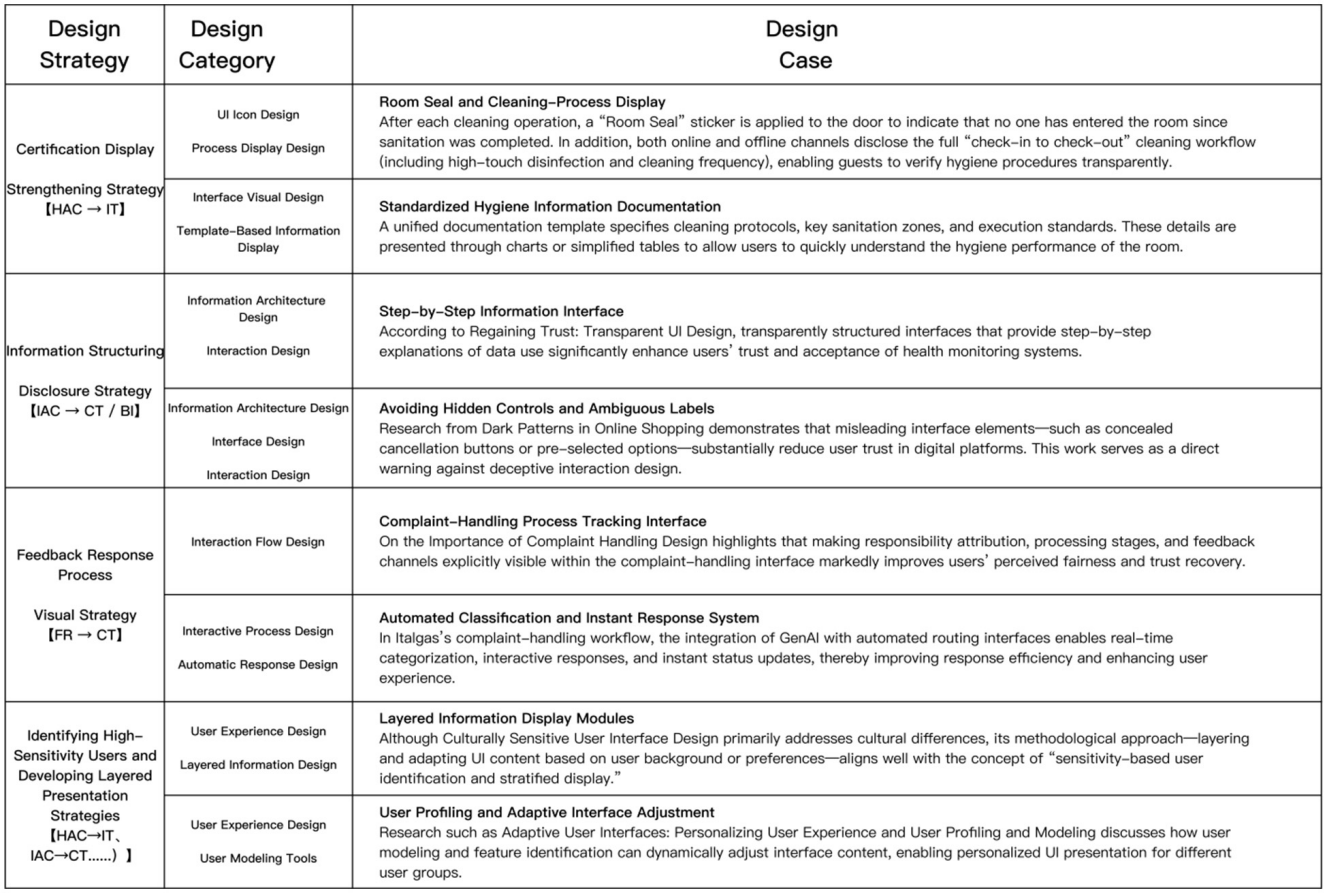
The framework summarizes four core trust-building strategies, their corresponding design categories, and illustrative cases/examples. These design interventions map to the hypothesized paths (e.g., HAC → IT, IAC → CT/BI, FR → CT) in the research model, demonstrating actionable methods to enhance perceived hygiene transparency, procedural fairness, and personalized user experiences.

All four strategies derive directly from the significant path and moderation analyses and are implementable within current platform UX patterns. Their core objective is to **reconstruct the hygiene–trust logic via visualization and interaction**, guiding users from uncertainty → perceived control → trust → behavioral conversion (Figure 6).

These findings are broadly consistent with prior research emphasizing the importance of transparency and institutional assurance in digital trust formation. However, compared with previous studies that primarily focus on general service quality perceptions, the present study highlights the specific role of hygiene governance mechanisms in shaping multidimensional trust within the hospitality platform context.

In conclusion, this study deepens the theoretical understanding of hygiene trust mechanisms in digital platforms by introducing objective trust measures, testing sensitivity-based moderation, and reflecting on non-significant paths. The findings provide practically actionable and locally adaptive insights for hotel and hygiene service platforms in designing information disclosure frameworks, hygiene governance systems, and user engagement strategies. Future trust mechanism development in digital platforms should continue to pursue institutional innovation, differentiated management, and technological enablement, to better address layered user cognition and behavioral diversity in an increasingly data-driven service environment. At the level of design practice, the proposed strategies extend the theoretical contributions by converting key trust-driving factors—platform hygiene certification, the authenticity and completeness of cleaning information, and the timeliness of feedback responsiveness—into interface elements that are visualized, interactive, and experientially perceivable. This approach enables the effective translation of institutional mechanisms into user-facing experience within digital hygiene governance. The governance and design implications discussed above are derived from the empirical patterns observed in this study and should therefore be interpreted as exploratory recommendations rather than direct policy prescriptions.

## 5 Limitations and future prospects

Although this study establishes a comprehensive “institutional mechanism—trust perception—behavioral intention” framework and systematically elucidates the key pathways and moderating effects of trust formation in digital platforms' hygiene management, several limitations remain that warrant future refinement and extension.

In terms of methodology, this research primarily relies on survey data and structural equation modeling (SEM). While these methods effectively uncover causal paths and significance differences among variables, the use of cross-sectional data restricts observation of the dynamic evolution of trust and longitudinal conversion from perception to behavior. Future studies could incorporate multi-source data, such as platform usage logs, behavioral trace analysis, or cont.

The sample was drawn predominantly from short-term accommodation users in mainland China, a group characterized by specific cultural norms, digital platform usage habits, and hygiene risk perceptions. These contextual features limit the generalizability of the findings. Extending comparative studies to Japan, South Korea, Europe, and North America—regions with more mature digital platforms and distinct institutional frameworks for hygiene governance—would strengthen the model's cross-cultural robustness and comparative validity.

Regarding variable design, although this study thoroughly examines the relationships between institutional mechanisms (e.g., certification, traceability, and disclosure) and user perceptions (e.g., cognitive and institutional trust), it omits potentially relevant dimensions such as affective trust and brand trust, which may further enrich the multidimensional structure of digital platform trust. Moreover, while Disgust Sensitivity (DS) demonstrated strong moderating effects, future work should decompose this construct into its subdimensions—such as disease avoidance, cleanliness preference, and need for control—to build a more psychologically grounded explanatory framework.

Although this study discusses several potential design approaches—such as visualized responsibility chains and layered information disclosure interfaces—these proposals are conceptual interpretations derived from the empirical relationships identified in the model. They should therefore be regarded as exploratory design implications rather than empirically validated interventions. Future research should employ A/B testing, prototype experiments, or eye-tracking studies to empirically examine how such designs affect users' cognitive load, perceived control, and behavioral conversion. Bridging theoretical mechanisms with verifiable design practices will not only enhance the applicability of trust research but also strengthen its design translation capacity, contributing to both academic and industry-level innovation in digital hygiene governance. Additionally, cross-cultural comparative studies are needed to test the robustness of the model across different institutional and cultural contexts.

## 6 Conclusion

From a theoretical perspective, this study examines hygiene information trust on hotel digital platforms by developing a behavioral intention model grounded in institutional mechanisms and mediated by user trust. The results suggest that institutional mechanisms—such as digital hygiene certification, information integrity disclosure, and responsibility traceability systems—are positively associated with higher levels of institutional trust reported by users in the surveyed platform context. In contrast, structured information disclosure and timely feedback responsiveness show stronger statistical associations with cognitive trust. At the behavioral level, both institutional trust and cognitive trust show positive associations with users' intention to stay in the sampled data. This pattern suggests that both institutional governance signals and users' cognitive evaluations may play complementary roles when users assess accommodation options on digital platforms. In addition, by incorporating disgust sensitivity as a moderating variable, the study introduces individual psychological differences into the analysis of platform trust formation, thereby enriching the theoretical understanding of hygiene-related perceptions in digital accommodation contexts. In addition, by incorporating disgust sensitivity as a moderating variable, the study introduces individual psychological differences into the analysis of platform trust formation, thereby enriching the theoretical understanding of hygiene-related perceptions in digital accommodation contexts.

From a practical perspective, based on the empirical patterns observed in this study, several design considerations are proposed to support the improvement of hygiene information presentation and governance transparency on digital platforms: (1) visual presentation of hygiene certification, (2) structured information disclosure, (3) process visualization of feedback responsiveness, and (4) identification and differentiated display for sensitive users. These considerations translate the observed statistical relationships into potential design implications that may assist platforms in communicating hygiene governance information more effectively.

Overall, this study contributes to the understanding of hygiene-related trust formation within the specific context of hotel digital platforms. The findings provide exploratory insights into how institutional information mechanisms may influence user perceptions in this environment. Future research should further deepen the analysis of behavioral mechanisms, empirically test the proposed design strategies, and promote the **integration of digital service ethics and hygiene governance** in the evolving landscape of digital platforms. It should also be noted that the findings are derived from the context of hotel digital platforms and may not be directly generalizable to other service sectors or cultural contexts.

Because the data were collected from users of hotel digital platforms within a specific survey context, the findings should be interpreted with caution and may not be directly generalizable to other service sectors, cultural environments, or offline hospitality settings.

## Data Availability

The raw data supporting the conclusions of this article will be made available by the authors, without undue reservation.
