# Correction: The effect of online hygiene information expression on customers' institutional and cognitive trust in tourist accommodation

**DOI:** 10.3389/fpubh.2026.1887030

**Published:** 2026-06-04

**Authors:** BoXi Zhang, Yuxuan Li, Xing Fang, Wei Huang, Qingshen Meng

**Affiliations:** 1 Wuhan University of Technology, Wuhan, China; 2 Huazhong University of Science and Technology Tongji Medical College Tongji Hospital, Wuhan, China

**Keywords:** digital platforms, hotel hygiene information disclosure, structural equation modeling, tourist accommodation, trust

Author BoXi Zhang was erroneously assigned as **corresponding author**. The correct **corresponding author** is Qingshen Meng.

The original version of this article has been updated.

